# Biological Amyloids Chemically Damage DNA

**DOI:** 10.1021/acschemneuro.4c00461

**Published:** 2025-01-09

**Authors:** Istvan Horvath, Obed Akwasi Aning, Sriram KK, Nikita Rehnberg, Srishti Chawla, Mikael Molin, Fredrik Westerlund, Pernilla Wittung-Stafshede

**Affiliations:** Department of Life Sciences, Chalmers University of Technology, 412 96 Gothenburg, Sweden

**Keywords:** amyloids, alpha-synuclein, DNA damage, catalytic activity, nanochannels, Parkinson’s disease

## Abstract

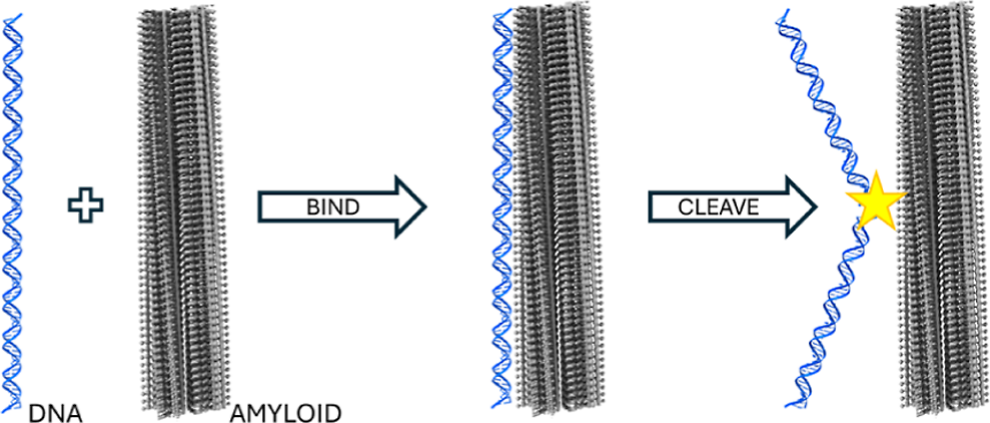

Amyloid fibrils are protein polymers noncovalently assembled through β-strands arranged in a cross-β structure. Biological amyloids were considered chemically inert until we and others recently demonstrated their ability to catalyze chemical reactions in vitro. To further explore the functional repertoire of amyloids, we here probe if fibrils of α-synuclein (αS) display chemical reactivity toward DNA. We demonstrate that αS amyloids bind DNA at micromolar concentrations in vitro. Using the activity of DNA repair enzymes as proxy for damage, we unravel that DNA-amyloid interactions promote chemical modifications, such as single-strand nicks, to the DNA. Double-strand breaks are also evident based on nanochannel analysis of individual long DNA molecules. The amyloid fold is essential for the activity as no DNA chemical modification is detected with αS monomers. In a yeast cell model, there is increased DNA damage when αS is overexpressed. Chemical perturbation of DNA adds another chemical reaction to the set of activities emerging for biological amyloids. Since αS amyloids are also found in the nuclei of neuronal cells of Parkinson’s disease (PD) patients, and increased DNA damage is a hallmark of PD, we propose that αS amyloids contribute to PD by direct chemical perturbation of DNA.

## 1 Introduction

Amyloids are long, ordered polymers of monomeric protein units noncovalently assembled through β-strands arranged perpendicularly to the fibril long axis forming a cross-β structure.^1^ The cross-β arrangement is the basis of all amyloid fibers, but the exact packing (fold, topology) of the β-strand arrangement in each perpendicular plane varies widely among amyloid systems; even the same polypeptide can adopt different amyloid polymorphs depending on conditions and other unknown factors.^2^ Many (maybe all) proteins can assemble into amyloids at extreme solvent conditions in vitro^1^ and, therefore, amyloid formation is viewed as an intrinsic property of polypeptide chains. Although several functional amyloids are known (e.g., bacterial curli),^3,4^ amyloid formation is mostly connected to human neurodegenerative diseases, such as Parkinson’s disease (PD) and Alzheimer’s disease, and type-2 diabetes.^5−8^ Here, proteins with normal functions as monomers start (for some unknown reason) to assemble into amyloids, resulting in both loss of monomer function as well as gain of toxicity coupled to the assembly processes. Today, we know of over 50 diseases linked to aberrant amyloid formation.^1^

Here we focus on the amyloidogenic protein in PD but, due to the general nature of amyloids, our observations may be extended to other amyloid systems. In PD patients, amyloid fibers of the synaptic, 140-residue protein α-synuclein (αS) accumulate in cytosolic inclusions, called Lewy bodies, in dopamine neurons along with death of such neurons in the substantia nigra.^9^ The cytoplasmic Lewy pathology is accompanied by genome instability in PD patients, animal models and cell cultures.^10−12^ Notably, most diseases involving amyloids include also genome instability as another hallmark. Although accumulation of DNA damage is recognized as a primary hallmark of general aging,^13^ PD patients and corresponding model systems display increased single-strand and double-strand DNA breaks.^11,14−16^ In fact, studies have demonstrated that increased DNA damage may be one of the earliest events detectable in neurodegenerative diseases such as PD.^15^

In addition to the cytoplasm, there is a significant fraction of αS in the cell nucleus, and functional roles in DNA repair, nucleocytoplasmic transport, and regulation of gene transcription have been proposed.^17−19^ However, most reports on nuclear αS suggest activities related to dysfunction.^12,20^ The amount of αS in the nucleus appears to be increased by αS post-translational modifications, αS pathological mutations, and chemical insults to the cells.^21−24^ Increased levels of αS in the nucleus were reported to perturb transcription of a master transcription activator^25^ and chromatin-bound αS was correlated with DNA breaks.^26^ Several studies have shown αS monomers to interact with DNA in vitro.^27,28^ We recently demonstrated that monomeric αS binding increases the persistence length of DNA,^29^ whereas binding of a truncated (pathological) variant of αS promotes DNA compaction.^30^ In addition to nuclear αS monomers, nuclear Lewy pathology, i.e., αS amyloids in the nucleus, has repeatedly been noted in PD patients as well as animal models.^31^ However, the consequences of αS amyloids in the nucleus remain unclear.^32−34^

Amyloid toxicity is often attributed to the ability to seed new amyloids, to translocate between cells, and to sterically block cellular functions. Amyloids have always been considered chemically inert until this was challenged when we showed that αS amyloids catalyze hydrolysis of ester and phosphoester bonds in vitro.^35,36^ In addition, we detected distinct chemical alterations of important metabolites in neuronal cell lysates (devoid of proteins; only small molecules present) upon incubation with αS amyloids.^37^ This enzyme-like behavior of αS amyloids, which has been paralleled by similar results on amyloid-β (linked to Alzheimer’s disease) and glucagon (hormone, unknown link to disease) amyloids,^38,39^ implies that many amyloid systems may have yet-unknown chemical reactivities.^35^

Here we test the hypothesis that αS amyloid interactions with DNA are directly responsible (at least in part) for the widespread DNA damage observed in PD patients. By combining in vitro bulk and single-molecule biophysical and biochemical experiments, we reveal that αS amyloids bind to double-stranded DNA with micromolar affinity. Such DNA-amyloid interactions result in both single- and double-strand DNA breaks. In support of biological relevance, DNA damage was found to be increased in yeast cells expressing human αS that had formed amyloids. We propose that DNA damage represents a toxic gain-of-function chemical activity of αS amyloids that contributes to disease progression.

## 2 Results

### 2.1 Amyloids of αS Bind DNA

**Figure 1:**
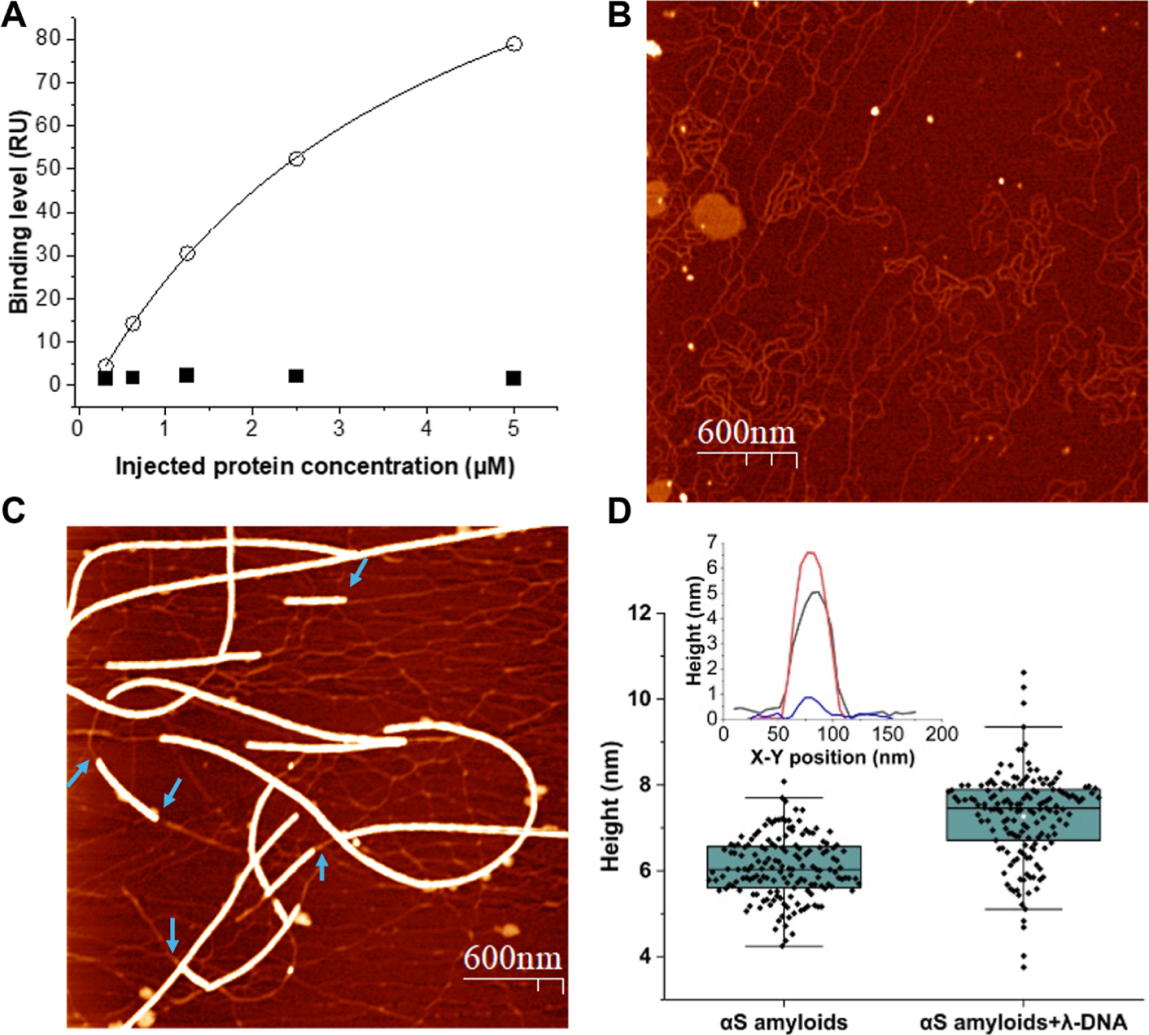
(A) Binding of monomeric (squares) and amyloid (circles) αS to immobilized DNA as measured by SPR, solid line shows hyperbolic fit. (B) AFM image of λ-DNA on mica surface. (C) AFM image of mixture of DNA and αS amyloids; blue arrows highlight where DNA appears to emerge after following along the amyloid long axis. *Z*-range for AFM images is 5 nm. (D) Box plot of height distribution of αS amyloids in the presence (average: 7.3 ± 1.0 nm) and absence (average: 6.1 ± 0.7 nm) of λ-DNA (*P* ≪ 0.0001). Inset shows an example cross section of λ-DNA (blue), αS amyloid alone (black) and αS amyloid with DNA (red).

To assess if αS amyloids can interact with double-stranded DNA (dsDNA), we employed surface plasmon resonance (SPR) analysis. αS in monomeric or amyloid form was injected in increasing concentrations over immobilized 50 base-pair, dsDNA molecules. The SPR response data show that αS amyloids (but not monomers) are capable of binding to the dsDNA at this condition (Figures 1A and S1). The apparent K_D_ estimated for the αS amyloid-DNA interaction is 4.0 μM ± 2.1 μM (using αS concentration in monomer units). This observation suggests that amyloid-DNA interactions may also occur in vivo as intracellular αS concentrations are estimated to be in the 5–50 μM range.^40^

To visualize the amyloid–DNA interactions, we used AFM to analyze incubated mixtures of dsDNA (here, λ-DNA, 48.5 kbp) and αS amyloids that had been deposited on mica surfaces. DNA alone showed (as expected,^41^) elongated curly structures with heights of 0.6–0.8 nm (Figure 1B). For mixtures of DNA and αS amyloids, DNA molecules were always found in proximity to the αS amyloids. The DNA molecules appeared to run along the amyloid fibril long axis for extended distances and then protrude and reach over to other amyloid fibrils, creating a network of DNA-bridged amyloids (blue arrows in Figure 1C, additional images in Figure S2). The αS amyloids appeared similar by AFM as to without DNA (Figure S2) but their heights increased by approximately 1 nm in the presence of DNA (Figure 1D) in accordance with most amyloids being covered by one or two dsDNA molecules.

### 2.2 Amyloids of αS Damage DNA

**Figure 2:**
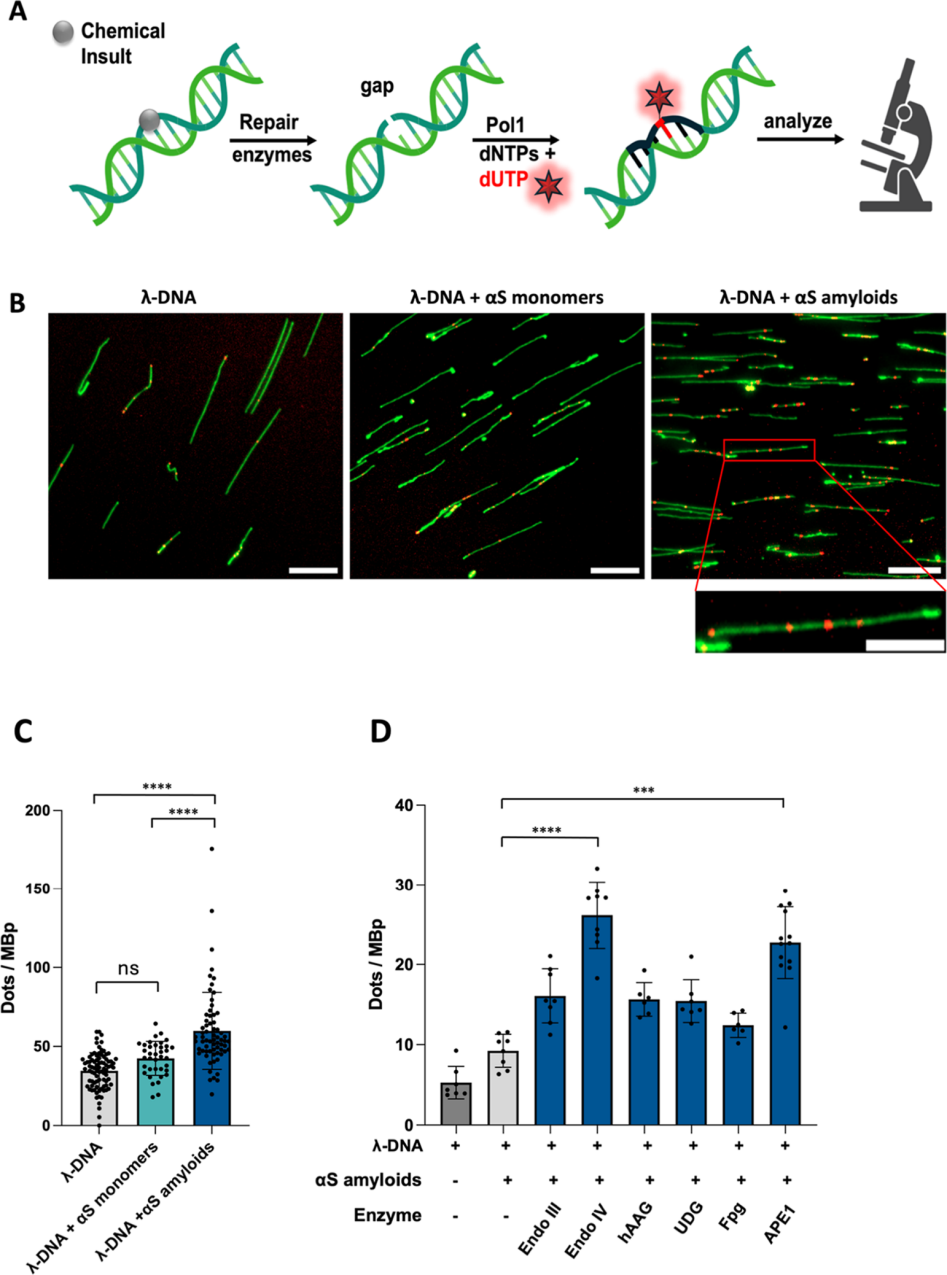
(A) Scheme of DNA damage detection. λ-DNA incubation with αS amyloids or αS monomers was followed by enzymatic repair and thereafter incorporation of fluorescent nucleotides at the damage sites. (B) Fluorescence microscopy image of labeled λ-DNA after incubation with αS monomers or amyloids and stretched on a functionalized glass coverslip. The DNA backbone was stained with YOYO-1 (green) and red dots are fluorescent nucleotides incorporated at damage sites. Scale bar = 10 μm. (C) DNA damage detection using a repair enzyme cocktail. Error bars indicate standard deviation calculated from biological replicates. (D) Detection of DNA damage using single repair enzymes. Error bars indicate standard deviation calculated from technical duplicates. *P*-values; ns, not significant; ***P ≤ 0.0002; ****P < 0.0001.

To investigate if the interaction between αS amyloids and dsDNA results in chemical perturbation of the DNA, we first used a single molecule imaging technique to assesses single-strand damage.^42−44^ After overnight incubation of αS amyloid and λ-DNA, the protein was removed and the formation of single-strand DNA lesions was probed with an enzyme cocktail of glycosylases and endonucleases (see Materials and Methods). These enzymes recognize different lesions (including oxidized bases, alkylated bases, nicks, abasic sites, and uracils) and then prepare the site for gap filling.^44,45^ By subsequent addition of DNA polymerase 1, and a combination of unlabeled dNTPs and fluorescently labeled aminoallyl-dUTP-ATTO-647N, damaged sites are repaired (Figure 2A). Each repaired lesion becomes fluorescently labeled because the polymerase is progressive and inserts many bases around the damage site of which likely at least one will be a labeled UTP. To quantify the number of DNA damage sites, the DNA backbone was labeled by YOYO-1, a bis-intercalating fluorescent dye commonly used to stain DNA,^46^ followed by stretching on silanized coverslips. The damage sites are observed as fluorescent “dots” along the stretched DNA molecules (Figure 2B). Upon quantifying detected damage sites on the DNA, we find a significant increase after incubation with αS amyloids as compared to dsDNA alone or upon incubation with αS monomers (Figure 2C). Similar results were obtained when amyloids of a C-terminally truncated αS form, αS(1–119), with 21 residues in the C-terminus removed, were incubated with DNA (Figure S3).

To assess what type of dsDNA damage the αS amyloids promote, the repair enzyme cocktail constituents were assessed one by one. From the results of such experiments (Figure 2D), we found Endo IV and APE1 enzymes to be most active, suggesting that the types of lesions they repair are what the αS amyloids are mostly causing. Endo IV and APE1 have endonucleolytic and phosphoglycolate activities; they often repair nicked, abasic and oxidatively damaged sites.^47−51^

Notably, the enzymes in the cocktail do not probe dsDNA breaks. However, the analysis also reveals the length of the DNA molecules on the coverslips, which can hint to putative double-stranded breaks (observed as shorter DNA molecules). Such analysis indicated shorter dsDNA molecules after αS amyloid incubation (Figure S4), but for more accurate analysis of DNA length changes, we turned to nanochannel studies in solution.

### 2.3 Amyloids of αS Cleave DNA

**Figure 3:**
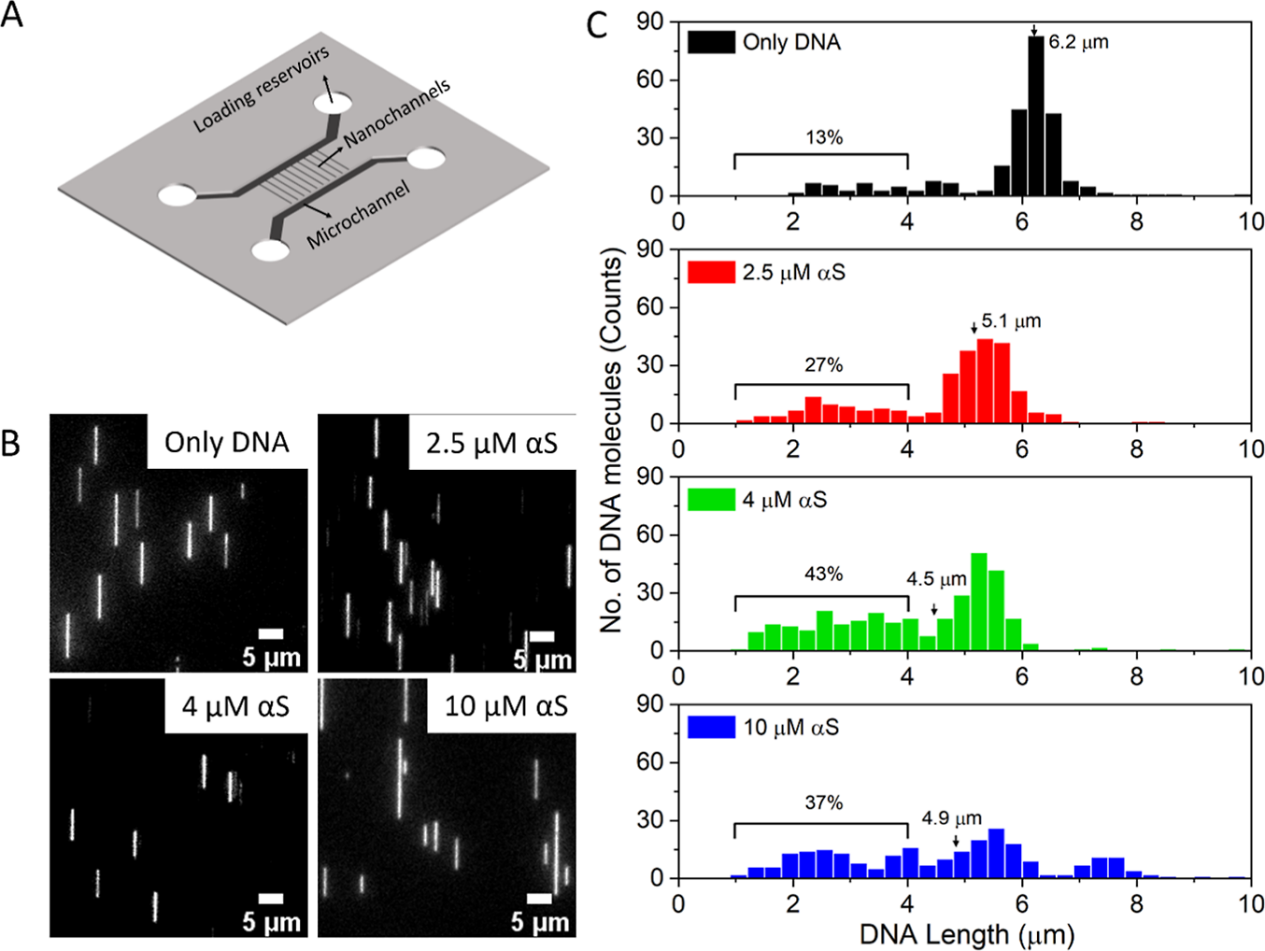
(A) Schematic of the nanofluidic device. (B) Fluorescence images of λ-DNA molecules after incubation (and removal) with 0 μM (control, only DNA), 2.5, 4 and 10 μM αS amyloids in the nanochannels. (C) Distribution of lengths of λ-DNA molecules in the nanochannels. Median length of DNA molecules (arrows) and percentage of molecules with lengths of 4 μm or less are indicated in each panel.

To investigate if αS amyloids can cause DNA double-strand breaks, we assessed the length of individual λ-DNA molecules in the presence of αS amyloids using nanofluidics (Figure 3A). The length measurements were again facilitated using YOYO-1. Samples of 5 μM λ-DNA (base-pair) mixed with varying αS amyloid concentrations (0, 2.5, 4, and 10 μM) were analyzed (Figure 3B). The results from length measurements of ∼250 DNA molecules for each amyloid concentration (Figure 3C) reveal that the median length of individual DNA molecules reduced from 6.2 μm (control) to 5.1 μm (2.5 μM αS amyloid) to 4.5 μm (4 μM αS amyloid). There is a considerable increase in the presence of very small DNA molecules (length below 4 μm) when αS amyloids are present (27% for 2.5 μM, 43% for 4 μM and 37% for 10 μM αS amyloid) as compared to DNA alone (13% shorter than 4 μm).

We exclude DNA compaction as the explanation for the observed shorter DNA for several reasons. First, if it had been DNA compaction, we would expect a gradual decrease in length for the whole population of DNA molecules. Notably, this is what was observed for the C-terminally truncated aS(1–97) monomer interacting with DNA (gradually shorter length for the DNA population, up to 25% length reduction, as a function of αS concentration) in an earlier study where DNA compaction was proposed.^30^ Instead, we here observe a wide span of lengths with many very short DNA molecules (lengths reduced by up to 75%) in the presence of αS amyloid fibrils, while some molecules remain intact. Second, if the very short DNA molecules we observe had been compacted DNA, they should collapse fully to a blob, not stop at a short but still elongated state.^52^ Many earlier studies have discussed how genomic DNA molecules adopt a toroidal shape when compacted in the presence of compaction agents.^53^ Such blob-like conformations have been shown in earlier nanochannel studies involving for example heat-stable nucleoid-structuring protein (H-NS) and dextran as compacting agents.^54^ Moreover, before complete compaction of a DNA molecule, the DNA is often partly compacted locally along the DNA, leading to varying dye emission intensity along the DNA due to variation in the local amount of DNA along the DNA contour.^55,56^ We do not observe this uneven behavior for the longer (but still shortened) DNA molecules. Based on these observations, we exclude DNA compaction and conclude that reduced lengths of the DNA molecules in the presence of αS amyloids is due to DNA fragmentation. DNA fragmentation may be a result of direct double-strand or single-strand breaks on opposite DNA strands close enough to each other to allow the molecule to break into two pieces with single-stranded overhangs on each end.

When the αS amyloid concentration was 10 μM, we observed DNA lengths varying between 1 and 8.5 μm, i.e., in addition to many short molecules we also detected some that appear longer than the median DNA length in the control (DNA only) experiment. Since we previously found (using the same method) that wildtype αS monomers increase the length of DNA molecules, we speculate that there is a small fraction of αS monomers in our samples that cause the extended DNA molecules. When αS amyloid formation reaches saturation, fibrils are in equilibrium with monomers. Reported values of the equilibrium concentration of monomers range between 0.7 and 28 μM.^57^ In our hands, typically 5–10% of the initial αS monomers remain as monomers after a completed aggregation experiment. Although we remove residual monomers before our experiments, the amyloids may shed some monomers during the incubation with DNA. At 10 μM αS amyloid concentration, the concentration of monomers in the sample may be sufficient to result in a detectable (but small) amount of extended DNA molecules.

### 2.4 Increased DNA Damage in αS Expressing Yeast

**Figure 4:**
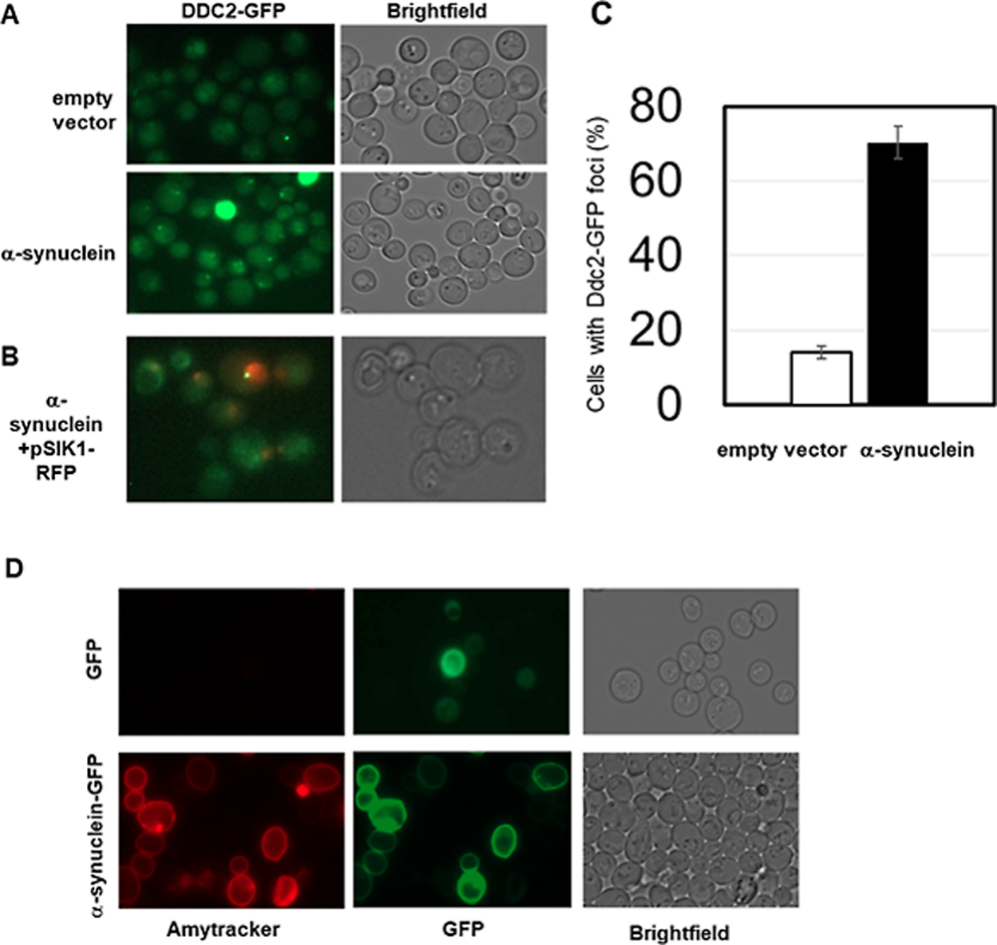
Analysis of DNA damage in actively growing yeast cells. Exponentially growing cells expressing the double-stranded DNA break sensor protein Ddc2 fused to GFP^58^ were imaged by fluorescence microscopy. (A) Cells were transformed with either the empty multicopy vector control plasmid (pYX242) or αS expressed from a strong, constitutive promotor. (B) To verify nuclear localization of Ddc2-GFP foci, cells were also transformed with a plasmid expressing a Sik1/Nop56-RFP fusion protein^71^ and imaged by fluorescence microscopy. (C) Quantification of the fraction of control and αS expressing cells displaying Ddc2-GFP foci. On average 14.1 ± 1.8 (5.6% SD) of control cells contained foci whereas 70.3 ± 4.3 (16.1% SD) of αS expressing cells contained foci. A two-sided and two-tailed *t*-test (*n* = 10 vs *n* = 14) indicates a statistically significant difference with *P* < 4.7 × 10^–10^. (D) Cells expressing GFP tagged αS or GFP only (green) from a strong constitutive promoter were stained with Amytracker (red) to assess presence of amyloids.

To assess αS-induced DNA damage in living cells, we turned to a yeast model system. In accord with the in vitro data, we find more DNA damage (detected by the double-stranded DNA break sensor protein Ddc2 labeled with GFP^58^Figure 4A) in actively growing cells expressing high levels of αS than in cells transformed with an empty vector. On average 14.1 ± 1.8% of control cells contained Ddc2 foci whereas 70.3 ± 4.3% of αS expressing cells contained Ddc2-GFP foci (Figure 4C). We confirmed the localization of these foci to the nuclei using the nucleolar/nuclear marker protein Sik1-RFP (Figure 4B).

Thioflavin T positive inclusions have been shown in yeast that expresses αS at high levels.^59^ In similarity, αS in our yeast system is expressed under the control of a strong constitutive promoter and from a multicopy plasmid. We directly confirmed the presence of amyloids in our yeast cells using the fluorescent amyloid-specific dye Amytracker in combination with GFP-tagged αS expression. The colocalization of Amytracker and αS signals inside the yeast cells confirms that the expressed αS indeed forms amyloids also in this model (Figure 4D).

## 3 Discussion

Here we report that αS amyloids interact with dsDNA and that such interactions result in chemical modification of the DNA. The amyloid structure is required for activity as the presence of αS monomers does not result in detectable DNA modifications. From a biophysical perspective, this adds a new activity to the repertoire of chemical reactivity that is emerging for biological amyloid fibrils.

**Figure 5:**
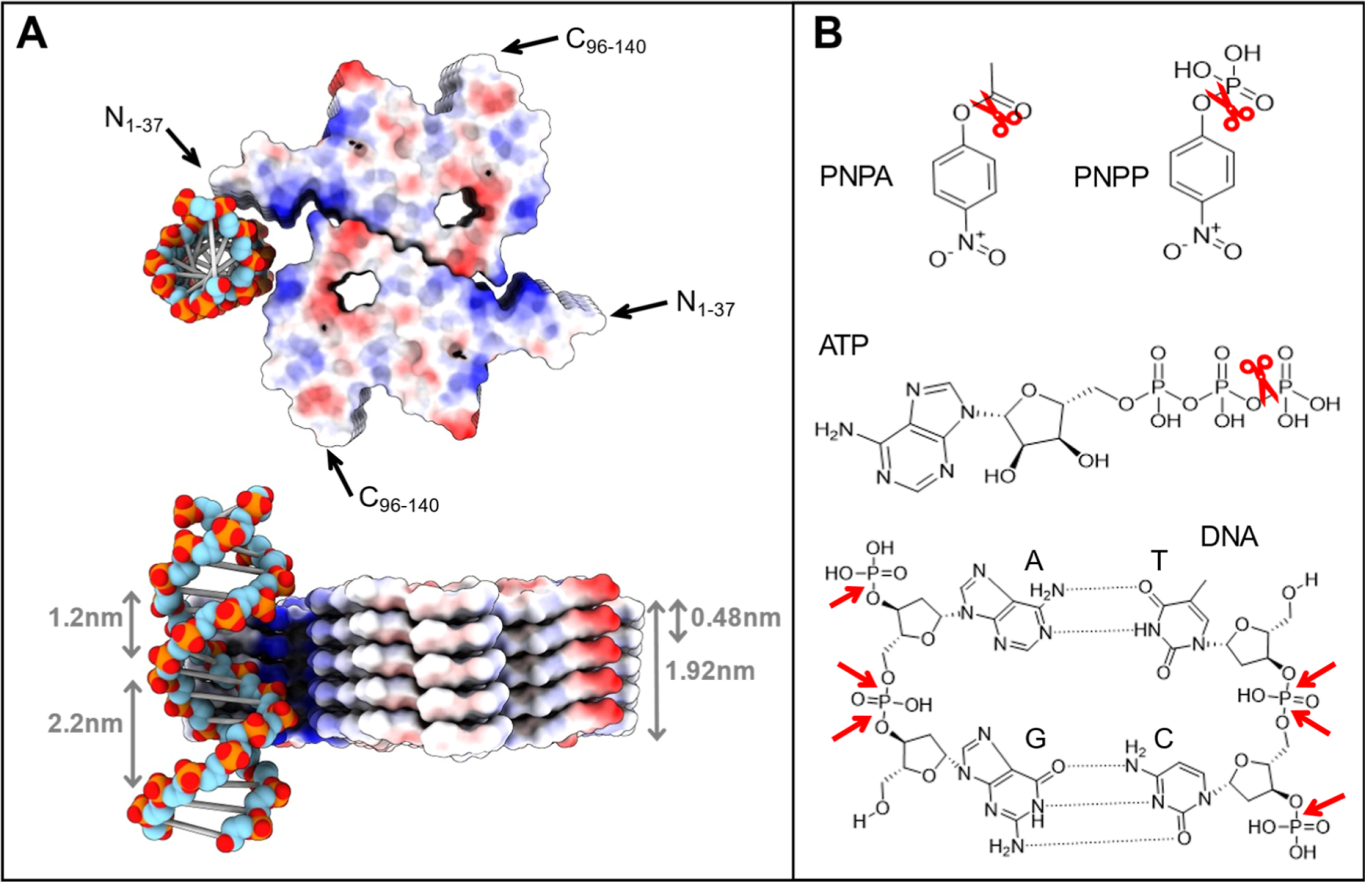
(A) Illustration of possible amyloid-DNA interaction. High-resolution structure of wild-type αS amyloid (6h6b) with 5 layers of monomers in two protofilaments is shown next to a piece of B-form DNA (3bse) positioned at the suggested interaction site near the protofilament interface (see text). The surface of the αS amyloid is colored according to electrostatics (blue, positive; red, negative); in the DNA, phosphorus is orange and oxygen is red. The positions where N- and C-termini disordered segments will extend from the ordered amyloid core are indicated. (B) Chemical structures of substrates (PNPA, PNPP, ATP, DNA; the latter two, this work) reported to be cleaved by αS amyloids so far. PNPA, *p*-nitrophenyl acetate (ester bond); PNPP, *p*-nitrophenyl phosphate (phosphoester bond). Phosphodiester bonds, proposed cleavage sites in DNA, are marked with red arrows in the DNA chemical structure. We note that other bonds in the DNA backbone may also be targets for the amyloid reactivity.

We recently reported catalytic activity of αS amyloids in vitro in the form of esterase and phosphatase activity on model ester and phosphoester substrates^35,36^ (substrates shown in Figure 5B). Similar activities have been reported for amyloid-β and glucagon amyloids; in addition, dephosphorylation of ATP (also shown in Figure 5B) was reported for the latter amyloid.^38,39^ Since the phosphate groups in the DNA backbone are exposed on dsDNA molecules, we speculate that αS amyloids damage DNA by phosphoester bond cleavage. If so, one expects αS amyloids, like glucagon amyloids, to hydrolyze ATP. Indeed, by the use of malachite green to detect inorganic phosphate,^60^ we observed a buildup of free phosphate when αS amyloids were incubated with ATP (Figure S5).

The αS amyloid structure has an ordered core with a repetitive surface pattern of identical residues running along the fiber long axis (Figure 5A). In surface cavities on the ordered amyloid structures, arrays of reactive sites may exist. Inspection of a typical wild-type αS amyloid structure (6h6b.pdb, there are several with similar overall fold) reveals a positive cleft with lysine residues running along the interface between the two protofilaments (Figure 5A, more structures in Figure S6). We propose that this region interacts with the negatively charged phosphates of the DNA backbone. When comparing dimensions, there will be one phosphate group of the twisting DNA helix directly facing the amyloid at every third (for minor groove) or fifth (for major groove) layer of the amyloid fibril (see Figure 5A, note that it is a hypothetical model only). Electrostatic attraction may pull on the DNA backbone toward the positively charged amyloid cleft. The affinity will be magnified due to the repetitive nature of both molecules and, at some places along the interface, protruding side chains on the amyloid surface may create sites that favor cleavage of DNA phosphodiester bonds (Figure 5B). Notably, the ordered αS amyloid core is surrounded by floppy N- (approximately 40 residues) and C-termini (approximately 45 residues) that do not adopt well-defined structures when analyzed by cryo-EM and other high-resolution methods (the positions of these extensions in the amyloid core are indicated in Figure 5A). Instead, they are thought to form a “fuzzy coat” surrounding the amyloid core. These peptides may affect interactions between the amyloid core and DNA; in fact, they could be responsible in full for the DNA interactions. Parts of the flexible N-terminus of αS harbor many positively charged side chains that may interact with DNA independently or provide stabilizing interactions around the DNA in addition to core amyloid interactions. In accord with termini contributions, another study showed that when a protein bound to the C-terminal floppy parts of αS amyloids, part of the αS floppy N-terminus folded onto the amyloid core.^61^ We did not find increased DNA damage activity by αS amyloids with a C-terminal truncation of 21 residues, implying that the floppy C-terminus does not block DNA interactions. However, many further studies of reaction mechanisms and substrate binding sites are needed to understand how αS amyloids chemically damage DNA on a molecular level. From a fundamental scientific view, the range of chemical reactivity that is harbored in biological amyloids (that can adopt many polymorphs of the common cross-β structure) may be vast and deserves exploration.

Although most of our experiments involve purified αS and DNA in vitro, the findings have biological significance as αS amyloids are found in nuclei of neuronal cells, along with widespread DNA damage, in PD patients and animal models.^15,16^ Many studies focus their investigations on Lewy body formation in the cytoplasm, but consistently report αS positive inclusions also in the nuclei.^32−34^ One study could demonstrate, using GFP-tagged aS, that nuclear αS amyloids are able to move between cells and enter nuclei of cells not expressing GFP-tagged αS.^31^ Since monomeric αS is present in the nucleus at normal conditions,^19^ and moves in and out in a dynamic fashion,^21,22^ amyloid formation may also be triggered directly in the nucleus from monomers residing there. Several in vitro studies have demonstrated that the presence of DNA can stimulate αS amyloid formation.^27,28^ Even if DNA is wrapped around histones and interacts with other proteins in nuclei, the DNA is exposed at transcriptionally active sites. In accord with our findings, Vasquez et al. showed nuclear localization of αS to be necessary for genome damage in cultured neurons.^26^

From a clinical perspective, amyloid formation in neuronal cell nuclei in PD patients may be destructive in two ways: by sequestering αS monomers and thereby blocking their proposed DNA repair activities^17^ (loss-of-function), as well as by inducing DNA chemical perturbation by direct DNA-amyloid interactions (toxic gain-of-function). αS amyloids may not only damage nuclear DNA: cytoplasmic αS amyloids may damage both RNA molecules and mitochondrial DNA, if mitochondrial membranes are perturbed. Indeed, mitochondrial dysfunction (which includes mitochondrial genome instability) is another signature of PD.^19,62^ Our work suggests that in addition to several reported toxic effects of αS amyloids, αS amyloids may also contribute to disease progression by direct chemical damage of DNA.

## 4 Materials and Methods

### 4.1 aS Expression and Purification

Wild-type and C-terminally truncated αS [αS(1–119); 21 C-terminal residues removed] was expressed in *Escherichia coli* grown in LB medium and purified using anion exchange chromatography and size exclusion chromatography as previously reported.^63^ Purified protein was stored at −80 °C. Before each experiment, gel filtration was performed to obtain homogeneous monomeric αS using a Superdex 75 10/300 (Cytiva, Uppsala, Sweden) column in TBS buffer (50 mM Tris, 150 mM NaCl, pH 7.6 at 25 °C, Medicago, Uppsala, Sweden).

### 4.2 Preparation of αS Amyloids

Amyloids of αS were prepared as described earlier.^36^ In short, freshly gel filtered monomeric αS was incubated with ∼5% premade amyloid fibrils for 5 days at 37 °C. Following incubation, samples were centrifuged at 13,400 rpm for 30 min. The pellet was resuspended in TE buffer (10 mM Tris, 1 mM EDTA pH 8.0). The amount of monomers that became amyloids were determined indirectly by measuring protein concentration left in the supernatant (as a measure of nonamyloid protein) using absorbance at 280 nm (extinction coefficient for αS of 5960 M^–1^ cm^–1^). In all experiments the concentration of amyloid fibrils denotes the monomer-equivalent concentration.

### 4.3 Surface Plasmon Resonance

Interactions between αS amyloids and DNA were studied using SPR on a Biacore X100 instrument with streptavidin coated sensor chip (Cytiva, Uppsala, Sweden). Amyloid fibrils of αS (prepared as above) were first sonicated to obtain shorter amyloids. Sonication was performed for 10 s using a probe sonicator (stepped microtip with Ultrasonic Processor Sonics Vibra-Cell; Sonics & Materials, Newtown, CT) running at 20% amplitude in an alternating cycle of 5 s (on mode) and 5 s (off mode). A 50-bp double-stranded biotin-labeled DNA (3′-CCTCTAGACCTGTACTACTCGAGAGATCGATCGACAGACGATGACTTAGC-5′) (Merck, Darmstadt, Germany) was immobilized on the sensor surface as described earlier.^64^ The level of immobilization was 200 RU. Single cycle measurements were performed by injecting increasing concentrations up to 5 μM of monomeric or fibrillar αS on the surface. Five M NaCl was used to regenerate the surface after each cycle. Background correction was done by subtracting the signal of the flow channel where no DNA was immobilized. The running buffer was HBS-P (10 mM HEPES, 15 0 mM NaCl supplemented with 0.002% P20 detergent) (Cytiva, Uppsala, Sweden). The dissociation constant was obtained by fitting of the binding levels at the end of the injection versus protein concentration data to a 1:1 binding model (using αS monomer concentrations) using evaluation software provided by the manufacturer (Cytiva, Uppsala, Sweden). The dissociation constant obtained is an average of 3 independent experiments.

### 4.4 Atomic Force Microscopy (AFM)

Prior to imaging, 50 ng/μL of λ-DNA (48.5 kb, Thermo Fisher, Waltham, MA, USA) in the absence or presence of 40 μM αS amyloids as well as αS amyloid fibrils alone, were incubated overnight at room temperature in TE buffer. Deposition of DNA and protein samples on mica surface were performed according to published guidelines.^65^ Freshly cleaved mica (Ted Pella Inc., Redding, CA, USA) was treated with 100 mM NiCl_2_ for 1 min and washed with Milli-Q grade water 3 times. The samples were diluted 10 times in 10 mM MgCl_2_, 25 mM KCl, 10 mM HEPES (pH 7.5) and incubated on the mica for 10 min followed by washing with Milli-Q grade water and drying with a gentle N_2_ flow. Images were recorded on an NTEGRA Prima setup (NT-MDT, Moscow, Russia) using a gold-coated single crystal silicon cantilever (NT-MDT, NSG01, spring constant of ∼5.1 N/m) and a resonance frequency of ∼180 kHz in tapping mode. 512 × 512-pixel images were acquired with a scan rate of 0.5 Hz. Images were analyzed using the WSxM 5.0 software. For the determination of αS amyloid heights, at least nine 5 × 5 μm images were taken in three different areas of the mica. The amyloid fibrils were automatically identified and average height of each individual fiber was measured using flooding analysis using the WSxM software.^66^ The presented data is based on 160 amyloid fibers for each condition.

### 4.5 DNA Damage Assay Using Repair Enzymes

50 ng/μL of λ-DNA in the absence or presence of 40 μM αS amyloid fibrils or monomers were incubated in TE buffer overnight at room temperature. The DNA was separated from the amyloids using the Genomic DNA Clean and Concentrator-10 kit (D4010, Zymo research) before labeling of damage sites. For this, 100 ng of the purified λ-DNA was incubated with a cocktail of repair enzymes which consists of 2.5 U each of APE1, Endo III, Endo IV, Endo VIII, hAAG, Fpg, and UDG, in 1× CutSmart Buffer (New England BioLabs) for 1 h at 37 °C. This was followed by incubation with dNTPs (1 μM of dATP, dGTP, dCTP, 0.25 μM dTTP (Bionordika Sweden) and 0.25 μM aminoallyl-dUTP-ATTO-647N (Jena Bioscience)) in 1× NE Buffer 2 (Bionordika Sweden) and 1.25 U DNA polymerase 1 (Promega) for 1 h at 20 °C. The reaction was terminated with 2.5 μL of 0.25 M EDTA (Sigma-Aldrich). Samples were stored at −20 °C until imaged on chemically modified glass coverslips.

Glass coverslips (18 × 18 mm^2^) were arranged in a coverslip rack and immersed in an acetone solution containing 1% (3-aminopropyl)triethoxysilane and 1% allyltrimethoxysilane (Sigma-Aldrich). After activation, the coverslips were rinsed with a (2:1 v/v) acetone/water solution and dried using N_2_ gas flow prior to DNA sample addition. Prior to analysis, the DNA samples were stained with 320 nM YOYO-1 (Invitrogen) in 0.5× TBE, supplemented with 2% β-mercaptoethanol (BME, Sigma-Aldrich) to prevent photobleaching, in a final volume of 50 μL. Next the DNA samples were added to the coverslips. To stretch the DNA, 3.2 μL of stained DNA sample was placed at the interface of a silanized glass coverslip and a clean microscopy slide (VWR).

Imaging of stretched DNA molecules were performed using an epifluorescence microscope (Zeiss AxioObserver.Z1) equipped with a Colibri 7 LED light source. For the DNA damage assay, the microscope was equipped with an Andor iXON Ultra EMCCD camera and 100× oil immersion objective. Band-pass excitation filters (475/40 and 640/30 nm) and bandpass emission filters (530/50 and 690/50 nm) were used for YOYO-1 and aminoallyl-dUTP-ATTO-647, respectively.

Data was analyzed with custom-made MATLAB software. DNA molecules were detected by the software to measure DNA length and count colocalized aminoallyl-dUTP-ATTO-647N labels (dots) along the DNA. The results were expressed as dots/μm. This was then converted to dots per megabase pairs (dots/Mbp) using a conversion factor of 3000 bp/μm estimated from stretching of intact λ-DNA molecules. Dots at ends of molecules, which could result from breaks during sample handling, and overlapping molecules were excluded. Damage, expressed as dots/Mbp, thus corresponds to the total number of damage sites detected per Mbp DNA.

To assess statistical significance, experiments were performed in biological replicates (4 and 2 for amyloid and monomer experiments, respectively) unless otherwise noted, and differences between groups were assessed by one-way Anova with Tukey’s multiple comparison test, with a family wise alpha threshold and confidence level of 95% (confidence interval). Total number of images analyzed for λ-DNA, λ-DNA + αS monomers and λ-DNA + αS amyloids were 85, 37 and 67, respectively, and at least 7000 DNA molecules (corresponding to over 300 Mbp) in total were analyzed for each condition. *P*-values are represented using the GraphPad Prism style; ns, not significant; ***P ≤ 0.0002; ****P < 0.0001.

### 4.6 Nanofluidic DNA Length Experiments

Lengths of individual DNA molecules as a function of added αS amyloids were measured by confining DNA in nanofluidic channels. For this, 5 μM (base-pair) λ-DNA was incubated with varying concentrations (0, 2.5, 4 and 10 μM) of αS amyloids in 1 × TE (10 mM Tris and 1 mM EDTA) buffer at room temperature for 4 h. After the incubation, YOYO-1 dye was added to the samples at 1:5 dye to base pair ratio and incubated at room temperature for 30 min. 3% (v/v) BME was added as an oxygen scavenger to suppress oxygen radical induced photodamage of the DNA.

The nanofluidic devices were fabricated in a cleanroom facility using standard semiconductor fabrication procedures, the details of which are described in detail elsewhere.^67,68^ Briefly, each device consists of two microfluidic channels that are 850 nm deep, and each microfluidic channel being connected to two sample loading reservoirs at its ends. The two microfluidic channels are connected by 200 parallel nanofluidic channels, with each nanofluidic channel being 150 nm in width, 100 nm in depth and 500 μm in length. The sample is loaded in one of the four loading reservoirs and the other three loading reservoirs are filled with buffer only. N_2_ pressure (2 bar) was applied to push the sample first from the loading reservoir into the microchannels and then into the nanochannels. DNA molecules are stretched in the nanofluidic channels due to nanoconfinement. For these experiments, the microscope described above was equipped with a Photometrics Evolve EMCCD camera, a 63× oil immersion objective and band-pass excitation (475/40 nm) and emission (530/50 nm) filters were used for YOYO-1 imaging. Using the imaging software ZEN, 20 subsequent images were recorded with an exposure time of 100 ms. Analysis of DNA lengths was performed using a custom-written MATLAB code after converting images to TIFF. Histogram plots (Figure 3C) were made using Origin Pro 2022b with *X*-axis as DNA length (μm) and *Y*-axis as number of DNA molecules (counts), with bin size of 0.3 μm.

### 4.7 DNA Damage in Yeast Cells

*Saccharomyces cerevisiae* yeast transformed with a multicopy plasmid expressing αS under the control a strong constitutive promoter^69^ was used in parallel with an empty-vector control strain (transformed with the empty vector pYX242). The proportion of cells exhibiting nuclear foci of the double-stranded DNA break sensor protein Lcd1/Ddc2 was compared for yeast with and without aS. For this, cells expressing a genomic Lcd1/Ddc2 GFP fusion protein were employed.^58,70^ Cells were grown overnight, diluted to OD 0.1, grown until in exponential phase (*A*_600_ = 1.2) and imaged using a Zeiss AxioObserver.Z1 inverted microscope equipped with Apotome/Axiocam 506 camera with a Plan-Apochromat 100x/1.40 Oil DIC M27 objective.

The percentage of cells containing Ddc2-GFP foci were evaluated in 16 different z-stacks per image. For empty vector yeast, a total of 854 cells were imaged in 3 independent experiments comprising in total 10 different images. For αS expressing cells, 1610 cells were imaged in 3 independent experiments comprising 14 different images. Cells were also transformed with the nucleolar/nuclear marker protein Sik1/Nop56-RFP^71^ and grown to exponential phase (*A*_600_ = 1.2) in synthetic defined (SD) glucose medium lacking uracil.^70^ Doubly labeled cells expressing αS were used to confirm nuclear localization (red, Nop56-RFP) of the Ddc2-GFP foci (green). To confirm amyloid formation of αS expressed in yeast, cells expressing αS-GFP or GFP only from a constitutive strong promoter (pRS426-GPD-αS-GFP or pRS426-GPD-GFP)^72^ were grown to midexponential phase and stained with 1:100 diluted Amytracker 680 (1 mg/mL dissolved in DMSO, Ebba Biotech, Stockholm, Sweden) for 6 h before visualized in the fluorescence microscope.

## Supporting Information Available

Figure S1 (SPR sensograms for binding), Figure S2 (additional AFM images of αS and DNA), Figure S3 [DNA damage induced by αS(1–119) amyloids], Figure S4 (DNA length distributions from analysis of DNA on coverslips), Figure S5 (ATPase activity for αS amyloids), and Figure S6 (high-resolution structures of the αS amyloid fold with putative DNA-binding site indicated) (PDF)
