# Supplementary material for: Biological Amyloids Chemically Damage DNA
Source: ACS Chem Neurosci. 2025 Jan 9;16(3):355–64. doi: 10.1021/acschemneuro.4c00461 (PMC11803820; doi:10.1021/acschemneuro.4c00461)
Supplement: Supplementary file 1 — cn4c00461_si_001.pdf [file cn4c00461_si_001.pdf]

Supporting Information

Biological amyloids chemically damage DNA

Istvan Horvath, Obed Akwasi Aning, Sriram KK, Nikita Rehnberg, Srishti Chawla, Mikael Molin, Fredrik Westerlund, Pernilla Wittung-Stafshede*

Content:

Figures S1-S6

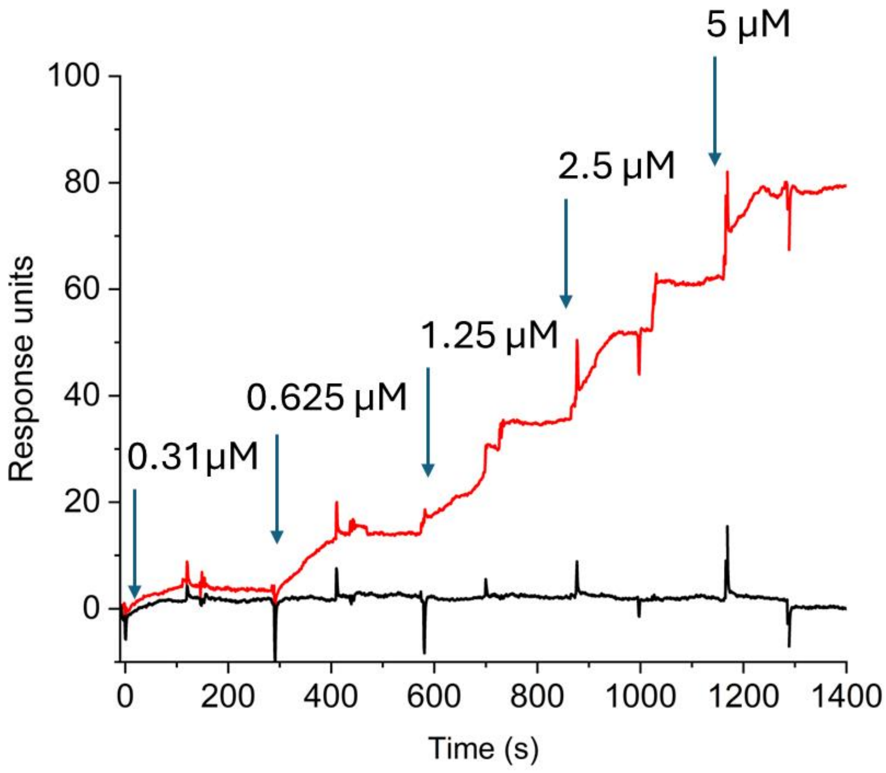

Figure S1. SPR sensogram measured in single cycle analysis mode. Black trace, injection of increasing concentrations of monomeric αS on DNA-coated surface; red trace, injection of the same protein concentrations (indicated in graph) of αS in amyloid form**.**

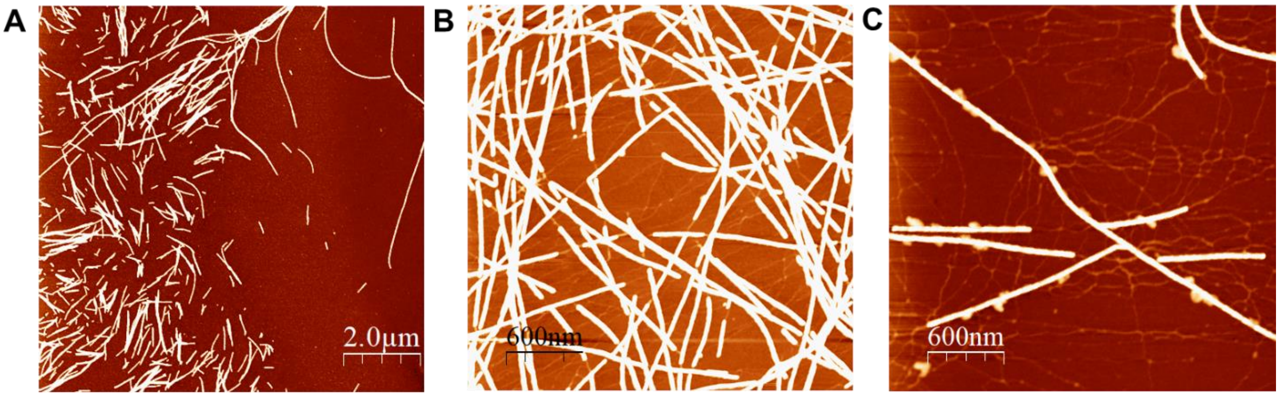

Figure S2. Atomic force microscopy image of αS amyloids alone (A) and when incubated with λ-DNA (B and C). Z-range set to 5 nm on all images.

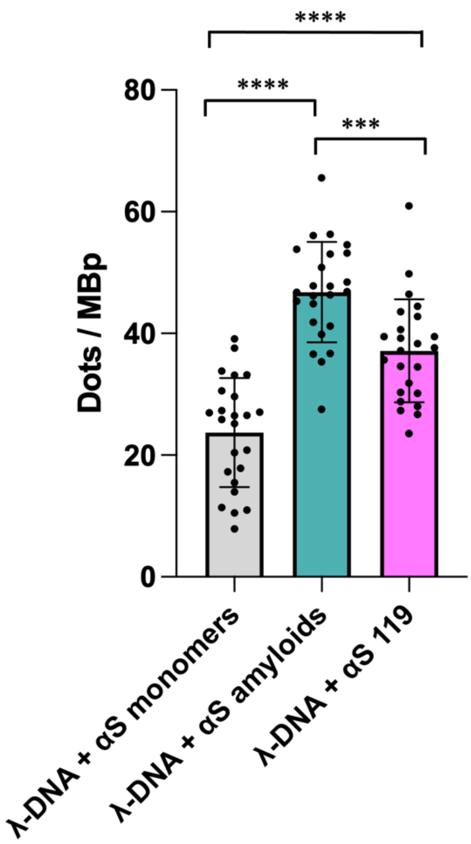

Figure S3. DNA damage detection using an enzyme cocktail in λ-DNA that was incubated with αS monomers or αS amyloids or C-terminally truncated αS(119). Error bars indicate standard deviation calculated from technical duplicates. P-values are represented using the GraphPad Prism style; ***$P \leq 0.0002$; ****$P < 0.0001$.

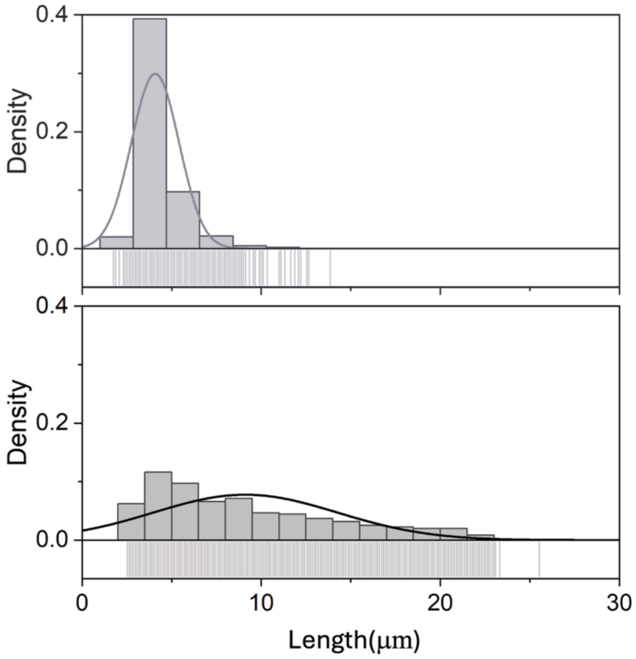

Figure S4. Frequency distributions of λ-DNA molecule lengths when alone (bottom panel) or after incubation with αS amyloids (top panel). From analysis of DNA stretched on coverslips (see **Figure 2**).

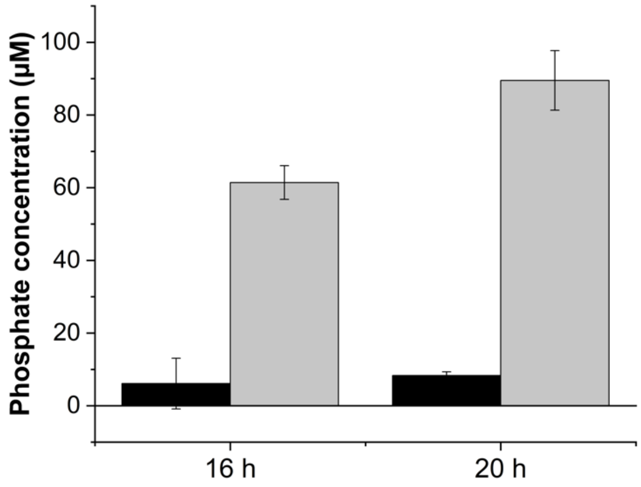

Figure S5. ATPase activity of αS amyloids detected as production of inorganic phosphate. After incubation of 1 mM ATP alone (black bar) and with 40 µM aS amyloids (grey bar) for 16 and 20 hours (at 37 °C in 20 mM Tris buffer with 5 mM $MgCl_2$ and 1 mM EGTA), the free phosphate concentration was determined via addition of malachite green (Phosphate assay kit, MAK-308, Merck, Darmstadt, Germany). The manufacturer's instructions were followed and absorption at 620 nm measured in 96 well plate using a BMG Fluostar Omega plate reader (BMG Labtech, Ortenberg, Germany). A phosphate standard series was used to convert the absorbance values to phosphate concentration. The zero timepoint was subtracted to allow calculation of amount phosphate generated during the incubation. Error bars are showing standard deviation of three replicates**.**

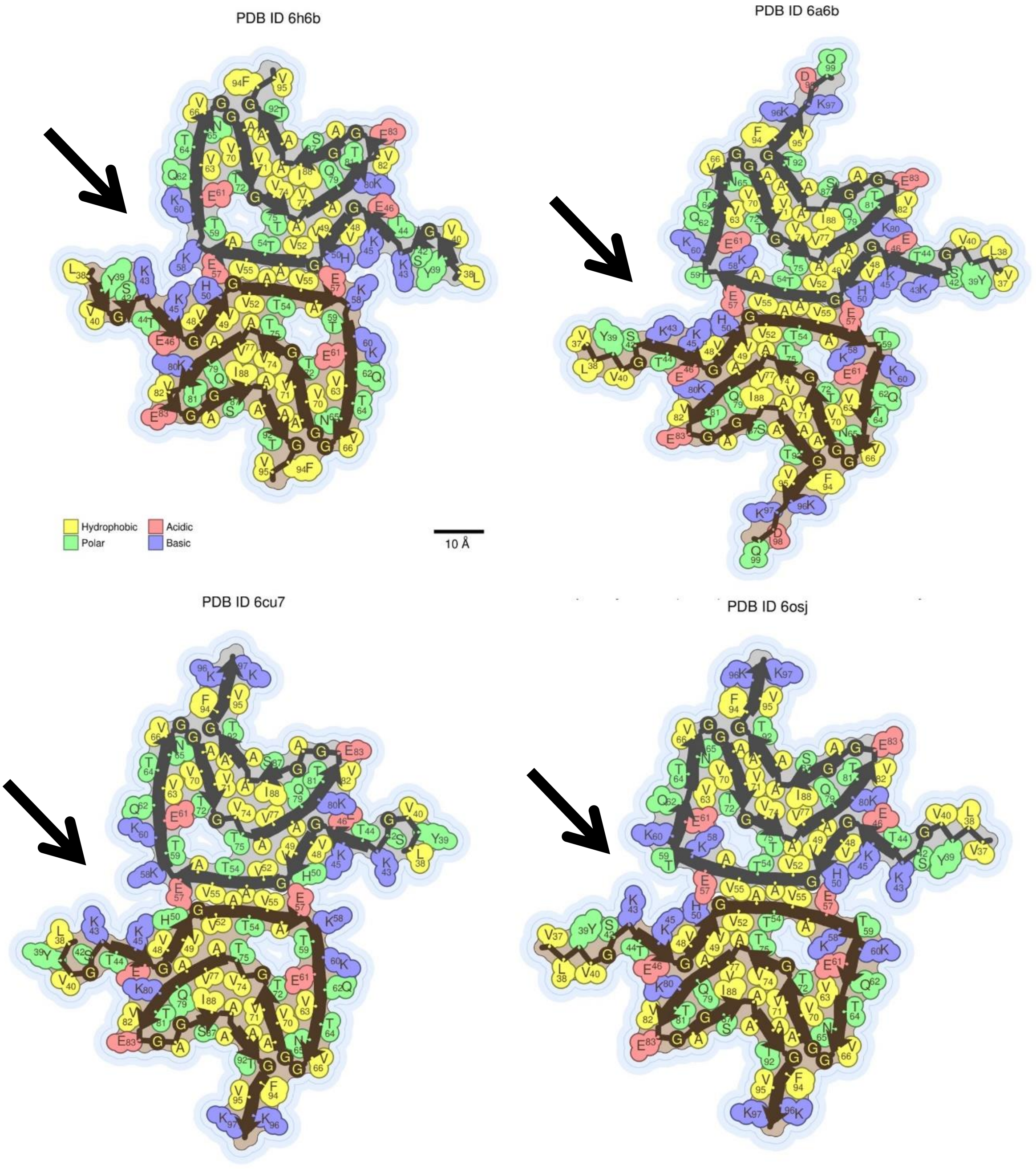

Figure S6. One layer of the cross-β fold of four representative wild-type αS amyloid high-resolution structures that include two protofilaments. Note the exposed lysines, K43, K45, K58 and K60, that seem to form a positive cluster (perhaps with H50) in a cleft near the interface between the two protofilaments (arrow on one side of each filament) in all of them. Images taken from the Amyloid Atlas at https://people.mbi.ucla.edu/sawaya/amyloidatlas/.
